# *Camellia oleifera* CoSWEET10 Is Crucial for Seed Development and Drought Resistance by Mediating Sugar Transport in Transgenic *Arabidopsis*

**DOI:** 10.3390/plants12152818

**Published:** 2023-07-29

**Authors:** Zhihua Ye, Bingshuai Du, Jing Zhou, Yibo Cao, Lingyun Zhang

**Affiliations:** Key Laboratory of Forest Silviculture and Conservation of the Ministry of Education, The College of Forestry, Beijing Forestry University, Beijing 100083, China; zhihuayepu@163.com (Z.Y.); dubingshuai624@163.com (B.D.); zhoujin.1234@163.com (J.Z.); caoyibo@bjfu.edu.cn (Y.C.)

**Keywords:** *Camellia oleifera*, sugar transport, SWEETs, seed development, drought tolerance

## Abstract

Sugar transport from the source leaf to the sink organ is critical for seed development and crop yield, as well as for responding to abiotic stress. SWEETs (sugar will eventually be exported transporters) mediate sugar efflux into the reproductive sink and are therefore considered key candidate proteins for sugar unloading during seed development. However, the specific mechanism underlying the sugar unloading to seeds in *Camellia oleifera* remains elusive. Here, we identified a SWEET gene named CoSWEET10, which belongs to Clade III and has high expression levels in the seeds of *C. oleifera*. CoSWEET10 is a plasma membrane-localized protein. The complementation assay of CoSWEET10 in SUSY7/ura3 and EBY.VW4000 yeast strains showed that CoSWEET10 has the ability to transport sucrose, glucose, and fructose. Through the *C. oleifera* seeds in vitro culture, we found that the expression of CoSWEET10 can be induced by hexose and sucrose, and especially glucose. By generating the restoration lines of CoSWEET10 in *Arabidopsis atsweet10*, we found that CoSWEET10 restored the seed defect phenotype of the mutant by regulating soluble sugar accumulation and increased plant drought tolerance. Collectively, our study demonstrates that CoSWEET10 plays a dual role in promoting seed development and enhancing plant drought resistance as a sucrose and hexose transporter.

## 1. Introduction

*Camellia* (*C.*) *oleifera*, also known as oil-tea camellia, is within the genus *Camellia* of the family Theaceae. *C. oleifera*, along with palm, olive, and coconut, is considered one of the world’s four woody oil plants due to the high content and quality of the oil produced from its seeds [1]. *Camellia* oil has a chemical composition that is low in saturated fats and high in unsaturated fatty acids like oleic and linoleic acids, hence it is also known as ‘Eastern Olive Oil’ [2]. As a kind of premium oil, *Camellia* oil is beneficial to human health as it lowers blood cholesterol and increases lipid resistance to oxidation [3,4]. At present, however, the low yield of *C. oleifera* seeds has not been able to meet the increasing demand for human consumption.

The increased yield potential, calculated by the proportion of total biomass assigned to harvestable organs, is attributed to the improvement in the allocation efficiency of photosynthate [5,6]. Sucrose is the predominant transport form of photoassimilates, therefore, an efficient way to increase yield is to distribute more sucrose from the photosynthetic leaves to the heterotrophic organs, like roots, flowers, seeds, etc. [7,8]. The allocation and short-distance transport of sucrose consists of two processes, including loading in source leaves and unloading into sink organs by the symplastic pathway, apoplasmic pathway, or a combination of both. The phloem loading through the apoplasmic pathway takes place in the following model: sucrose is exported by SWEETs (sugars will eventually be exported transporters, one of the sugar transporters) from phloem parenchyma cells and is then transported by SUTs (sucrose transporters) into the sieve element–companion cell complex (SE/CC) [9]. After long-distance translocation in the phloem, sucrose can be imported into sink organs through the apoplasmic phloem unloading pathway: sucrose could be diffusely unloaded from CCs to the apoplast, then be immediately imported to the parenchyma cells of sink organs by SUTs, or be catabolized to hexoses, which are subsequently imported by SWEETs or other monosaccharide transporters [10]. Therefore, SWEETs play a role in the assignment of photosynthate into harvestable organs during plant development.

The SWEET family in plants has been identified as an unusual category of conserved sugar transporters and is found in almost all plants [11,12]. The conserved domain of SWEETs is the PQ-loop repeat, also commonly known as the MtN3/saliva (MtN3_slv) domain. SWEETs contain seven transmembrane domains (TMs) in eukaryotes, according to calculations [12,13], and have been phylogenetically classified into Clade I, II, III, and IV [11,14]. In particular, Clade III SWEETs mediate photosynthate partitioning into sink organs, such as the fruits and seeds of globally important crops, which determines the sugar content of fruits, seed size, and the content of seed carbohydrate, protein, and oil [15,16,17,18]. In fruits, SWEETs could affect sugar accumulation by mediating the membrane transport of sugars. The grape *VvSWEET10* and *VvSWEET15* are highly expressed in fruits and transport hexose during the period of fruit ripening, thus affecting the sugar content of grape berries [19,20]. The fruit growth of the tomato *SlSWEET15* is greatly influenced by transferring sucrose during phloem unloading and subsequently importing it into storage cells [21]. For seeds, SWEETs may support embryo development by catalyzing sucrose or hexose efflux and importing it into the developing seeds of many species. The *AtSWEET11*, *12,* and *15* in *Arabidopsis*, which imports sucrose from the seed coat or endosperm into the embryo, have an effect on embryo and seed growth by increasing the starch and lipid accumulation [22]. *ZmSWEET4c* in maize and *OsSWEET4*, its ortholog in rice (*Oryza sativa*), affect seed size by transporting hexose across the basal endosperm transfer layer in the period of grain filling [15]. The rice *OsSWEET11*, *14*, and *15* mediate sucrose transport in seeds during the grain filling period [17,23]. Thus, these studies reveal the possibility that SWEETs may make a difference in sugar transport in sink organs, especially seeds.

Despite their function in the growth and development of plants, there have also been numerous reports demonstrating that SWEETs play a key role in improving plant disease resistance by modulating plant–pathogen interactions [24,25,26,27]. Subsequently, as research on SWEETs intensified, more reports revealed their role in response to abiotic stress. It is the high level of soluble sugars, like sucrose and glucose, that contributes to the resistance to abiotic stress [28]. They act as osmoprotectants and antioxidants, interacting with the lipid bilayer to stabilize the membrane structure [29]. SWEETs located in the plasma membrane and tonoplast can regulate sugars [30]. As a result, these sugar transporters can promote plant adaptation to adverse circumstances, including cold stress, salt stress, drought stress, and others. So far, SWEET genes associated with drought stress tolerance have been successively found in *Arabidopsis* and rice [31,32]. In *Arabidopsis*, the expression of *AtSWEET11* and *AtSWEET12* is highly induced to enhance the transport of sucrose from the leaves to the roots under drought stress [33], while *AtSWEET17* is essential for drought tolerance by transporting fructose to modulate root growth and architecture [31]. In rice, the transcript abundance of *OsSWEET13* and *OsSWEET15* significantly increased under drought treatment [32]. However, the report regarding SWEETs involved in drought stress is very limited.

There have been some reports about the sugar unloading pathways related to CoSUTs in *C. oleifera* fruits [34,35]. However, SWEETs in *C. oleifera* have rarely been reported so far. In this study, we found that CoSWEET10, as a homolog of *AtSWEET10*, had high expression levels in the *C. oleifera* seeds. As a sucrose and hexose transporter, CoSWEET10 might also be feedback regulated by sucrose and hexose. Phenotypic experiments showed that CoSWEET10 could affect the development and sugar accumulation of seeds and enhance the drought tolerance of seedlings in transgenic *Arabidopsis*. These findings reveal the functions of CoSWEET10 and provide theoretical support for increasing seed yield and adaptation to the adverse environment of *C. oleifera*.

## 2. Results

### 2.1. Identification and Phylogenetic Analysis of CoSWEET10

**Figure 1:**
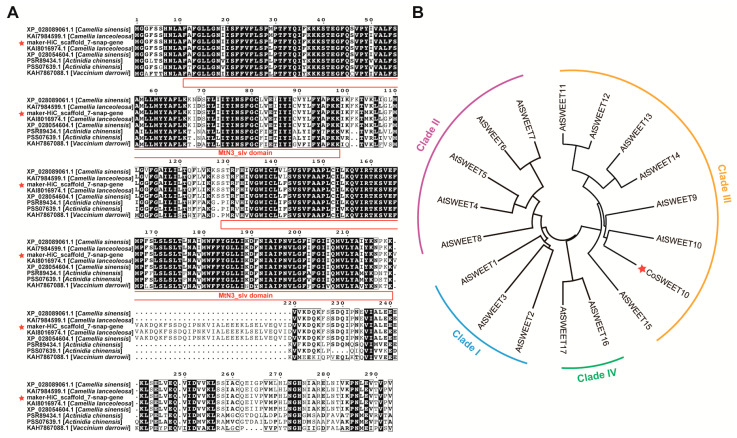
Protein sequence alignment of SWEETs and phylogenetic tree analysis. (**A**) Sequence properties of SWEETs. The two conserved MtN3_slv domains are indicated with red lines. (**B**) Neighbor-joining phylogenetic tree of SWEETs in *C. oleifera* and *Arabidopsis*. Place of CoSWEET10 in phylogenetic tree is marked with the red star. The amino acid sequences used for phylogenetic analysis are listed in Supplementary Table S2.

We characterized a SWEET family gene with high expression levels based on the transcript profile analysis of *C. oleifera*. To identify the underlying features of this gene, we obtained several genes belonging to the *SWEET* family from different plant species via BLAST against our gene sequence. The CDS length of our gene is 984 bp and its corresponding protein contains 327 amino acids (Tables S1 and S2). All of these proteins contain two conserved MtN3/saliva (MtN3_slv) domains which are the characteristics of SWEET proteins, and these two conserved domains’ positions were at the 11–98 and 129–219 amino acids, respectively (Figure 1A). Subsequently, this *CoSWEET* gene was named CoSWEET10, according to its *Arabidopsis* homolog, while it was located together with *Arabidopsis AtSWEET10* and was found to belong to Clade III through a phylogenetic tree (Figure 1B and Figure S1).

### 2.2. Expression Pattern and Localization of CoSWEET10

**Figure 2:**
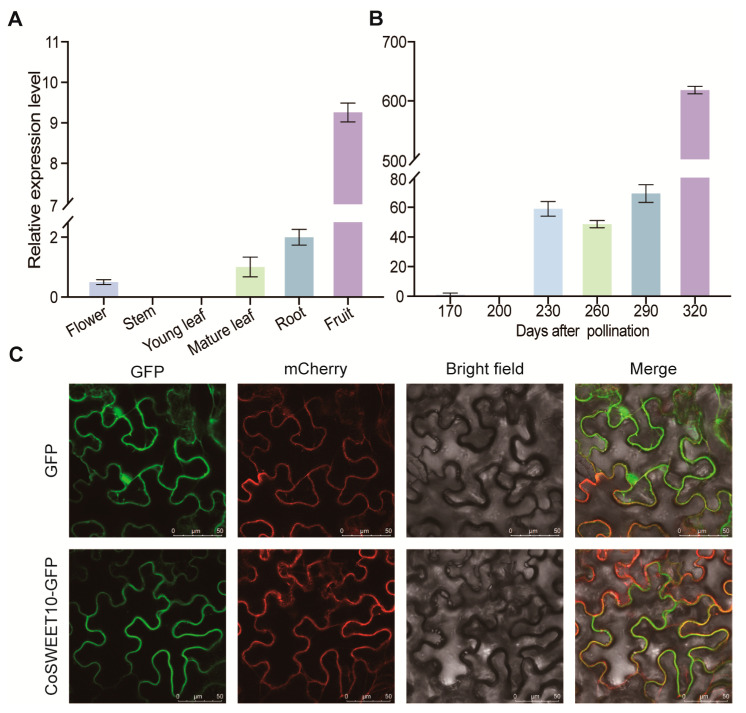
The expression pattern and localization of CoSWEET10. (**A**) The relative expression of CoSWEET10 in various tissues of *C. oleifera*. (**B**) The relative expression of CoSWEET10 in *C. oleifera* fruit from 170 to 320 DAP (days after pollination). The gene expression levels were determined by qRT-PCR and relative to GAPDH. The data are shown as the means ± SDs of three independent replicates. (**C**) The subcellular localization of CoSWEET10. GFP (empty vector) or CoSWEET10-GFP were transiently co-expressed with pBI121-mCherry-fABD2 in the epidermal cells of *N. benthamiana* leaves. The empty vector was a positive control. The pBI121-mCherry-fABD2 was a plasma membrane marker. Scale bars = 50 μm. The experiment was performed three times, each showed the same result.

To determine the expression pattern of CoSWEET10, we performed qRT-PCR on cDNA isolated from several different oil tea tissues, including flowers, stems, young leaves, mature leaves, roots, and fruits from field-grown plants in Changsha. The transcript level of CoSWEET10 was much higher in fruits than in other tissues, including flowers, mature leaves, and roots, where CoSWEET10 was slightly expressed (Figure 2A). Therefore, we further performed an expression pattern analysis of CoSWEET10 at different developmental stages of fruit. CoSWEET10 was highly expressed at 230 DAP (days after pollination) and peaked at 320 DAP when the expression level was about 10-fold as before (Figure 2B). These results imply that CoSWEET10 may be essential for fruit development, especially at the late stage.

To determine the subcellular localization of CoSWEET10, we transiently expressed it as a translation fusion to a green fluorescent protein (GFP) driven by the CAMV35S promoter (pCAM35S::CoSWEET10-GFP) in tobacco (*Nicotiana benthamiana*) leaves, while a standard plasma membrane marker protein (pBI121-mCherry-fABD2) was co-expressed together. The green fluorescent signal derived from CoSWEET10-GFP was clearly present on the plasma membrane and largely overlapped with the red fluorescent of PM-mCherry (Figure 2C). This result indicates that CoSWEET10 is a plasma membrane-localized protein.

### 2.3. CoSWEET10 Transport Substrate Specificity in Yeast Cells and Sugar-Induced Analysis in C. oleifera

**Figure 3:**
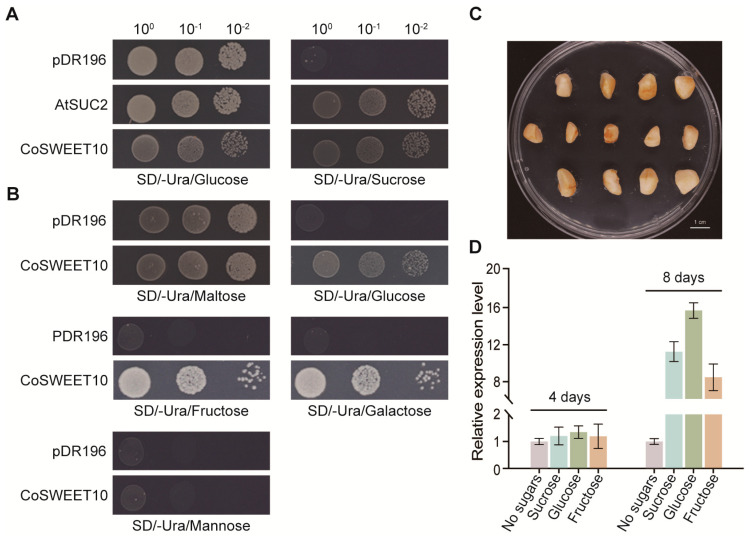
The transport activity of CoSWEET10 in yeast and sugar sensibility of CoSWEET10 in *C. oleifera* seeds. (**A**) The complementation assays of sucrose transport deficiency by CoSWEET10 in yeast mutant strain SUSY7/ura3. AtSUC2 was a positive control and empty vector pDR196 was a negative control. SD/−Ura solid media were supplemented with 2% glucose and 2% sucrose, respectively. (**B**) The complementation assays of hexoses transport deficiency by CoSWEET10 in yeast mutant strain EBY.VW4000. Empty vector pDR196 was a negative control. SD/−Ura solid media were supplemented with 2% maltose, 2% glucose, 2% fructose, 2% galactose, and 2% mannose. (**C**) An image of *C. oleifera* seeds cultured in vitro on media. Scale bar = 1 cm. (**D**) The relative expression levels of CoSWEET10 in *C. oleifera* seeds cultured in vitro on a medium containing different sugars. The seeds were collected after 4 d and 8 d cultures. Transcript levels were determined by performing qRT-PCR relative to GAPDH. The expression level of seeds cultured on media with no sugars was normalized to 1. The data are shown as the means ± SDs of three independent technical replicates.

To provide a functional characterization of CoSWEET10, we cloned its CDS into the pDR196 vector and transformed it into the yeast mutant strains, SUSY7/ura3 and EBY.VW4000, respectively. SUSY7/ura3 is a sucrose uptake-deficient yeast strain that is short of endogenous sucrose transporters and is capable of utilizing sucrose just absorbed by foreign sucrose transporters. It can grow only on glucose-containing media. EBY.VW4000 is a hexose uptake-deficient yeast strain that can merely utilize hexose absorbed by foreign hexose transporters and can grow only on media containing maltose, but not other monosaccharides. Yeast cells transformed with empty vector pDR196 acted as a negative control, while AtSUC2 was used as a positive sucrose transporter control. CoSWEET10 recovered the growth of SUSY7/ura3 on SD/−Ura media containing sucrose, similar to AtSUC2 (Figure 3A). The expression of CoSWEET10 also complemented the growth deficiency of EBY.VW4000 on SD/−Ura medium containing glucose, fructose, and galactose, but not mannose (Figure 3B). These results indicate that CoSWEET10 functioned as a specific transporter of sucrose, glucose, fructose, and galactose.

To further explore the influence of different sugars on the expression levels of CoSWEET10 in *C. oleifera*, we conducted in vitro seed cultivation. After disinfecting the seeds of *C. oleifera* at 230 DAP, we placed them on different solid mediums containing sucrose, glucose, and fructose (Figure 3C). Solid media containing no sugars was as a control. After 4 d and 8 d in the dark at 24 °C, seeds from the same treatments were pooled together. Then, we performed qRT-PCR on cDNA-isolated samples as described above. Compared with the control, CoSWEET10 transcript abundances of seeds that were cultured in vitro on a medium containing glucose, sucrose, and fructose, were all significantly increased at 8 d, while there was only a slight increase at 4 d (Figure 3D). Specifically, at 8 d, compared with the control, the CoSWEET10 expression level was ~16-fold higher when the seeds were cultured in vitro on a glucose-containing medium, followed by ~11-fold on a medium containing sucrose and ~8.5-fold on a medium containing fructose (Figure 3D). Therefore, these results intimate that CoSWEET10 expression could be induced by glucose, sucrose, and fructose.

### 2.4. Identification of Potential Self-Interaction of CoSWEET10

In general, some SWEETs need to form homo- or hetero-oligomers to perform sugar transport activity [36]. We speculated that CoSWEET10 could assemble homo-oligomers through self-interaction and thereby become functional. To verify this speculation, we performed bimolecular fluorescence complementation (BiFC) assays. The NH2-proximal half of YFP (nYFP) and C-proximal half of YFP (cYFP) were fused to the C-termini of CoSWEET10. Then, these two kinds of fusion proteins were transiently co-expressed in tobacco leaves. However, there was no homo-oligmerization observed in these assays (Figure S2). This result indicates that CoSWEET10 could not form homo-oligomers.

### 2.5. CoSWEET10 Functions as Sucrose and Hexose Transporter in Arabidopsis

**Figure 4:**
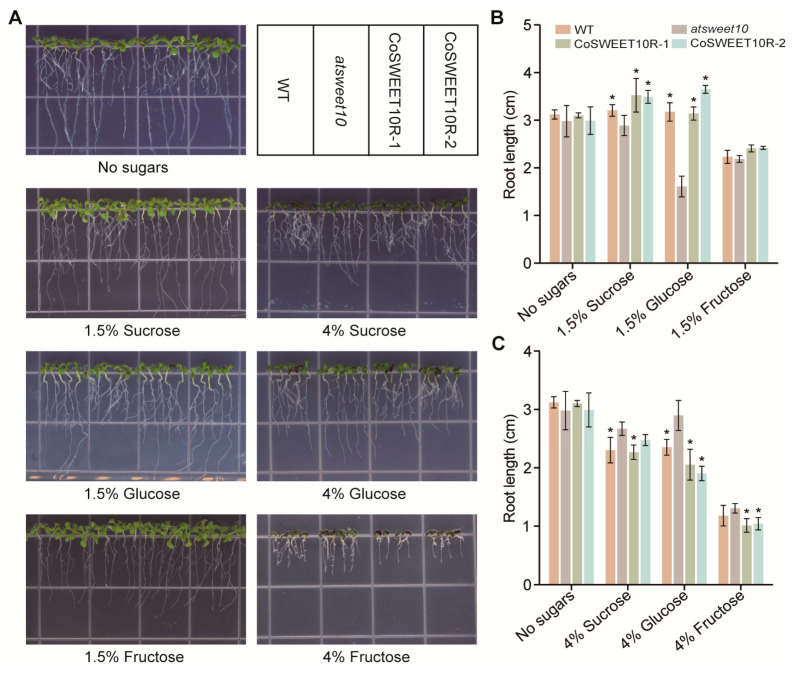
Functional analysis of sugar transport of CoSWEET10 in transgenic *Arabidopsis*. (**A**) The phenotypes of *Arabidopsis* seedlings of wild type (WT), *atsweet10* mutant, and two restoration lines: CoSWEET10R-1, and CoSWEET10R-2 grown on 1/2 MS containing different sugars with 1.5% and 4% concentrations. (**B**,**C**) The root length of *Arabidopsis* seedlings grown on 1/2 MS containing 1.5% sugars (**B**) and 4% sugars (**C**). The data represent the means ± SDs of four biological replicates. Significant differences from *atsweet10* under the same treatment are indicated by asterisks (* *p* < 0.05) according to Student’s *t*-test.

To further confirm the function of CoSWEET10 and its effect on plant growth, we grew *Arabidopsis* WT plants, *atsweet10* mutant plants, and two transgenic *Arabidopsis* restoration lines of CoSWEET10 for *atsweet10* mutant (named CoSWEET10R-1 and CoSWEET10R-2) (Figure S3) for 7 d on half-strength MS (1/2 MS) medium supplemented with 1.5% or 4% of sucrose, glucose, and fructose (Figure 4A). A 1/2 MS without any sugar was used as a control. On the 1/2 MS supplemented with 1.5% of sucrose or glucose, the root length of WT, *atsweet10*, R1, and R2 plants had the same tendency: R1 and R2 were obviously longer than *atsweet10*, which were significantly shorter than WT (Figure 4A,B). Especially on the 1.5% glucose-medium, the root length of *atsweet10* was almost half of that of WT, R1, and R2 lines, suggesting CoSWEET10 can restore the phenotype of mutant (Figure 4B). On 1/2 MS containing 4% sucrose or glucose, where the seedling growth had been suppressed to varying degrees compared to control, *atsweet10* had the least suppression, followed by WT, while R1 and R2 were the most inhibited (Figure 4A,C). Especially on 4% glucose-medium, the root length of R1 and R2 was distinctly shorter than that of *atsweet10* (Figure 4C). These results indicate that CoSWEET10 mainly functioned in the transport of glucose and sucrose. According to a previous report, fructose inhibited the growth of primary roots of the wild-type *Arabidopsis* but stimulated the formation of lateral roots [31]. Consistent with this report, our result showed that the growth of all seedlings was significantly inhibited on 1.5% and 4% fructose medium compared to the control (Figure 4A–C). The seedlings of R1 and R2 had more lateral roots on the 1.5% fructose-medium when compared to *atsweet10*, and the root length of R1 and R2 was obviously shorter than *atsweet10* on the 4% fructose-medium (Figure 4C). Thus, we could not exclude that CoSWEET10 was likely able to transport fructose as well.

### 2.6. CoSWEET10 Is Essential for Seed Development in Transgenic Arabidopsis

**Figure 5:**
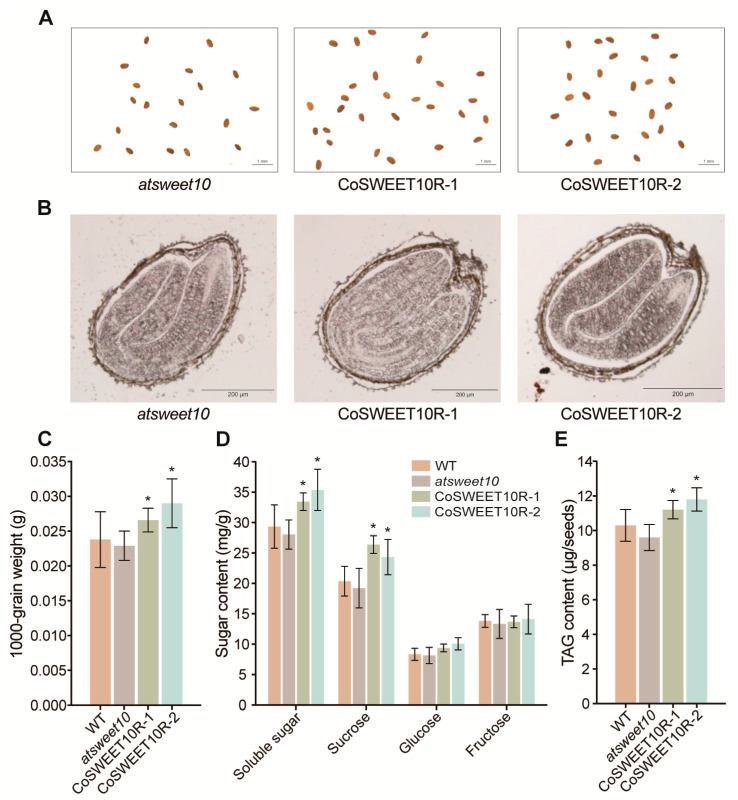
CoSWEET10 affects seed development in transgenic *Arabidopsis*. (**A**) The morphological phenotypes of mature *Arabidopsis* seeds of *atsweet10*, CoSWEET10R-1, and CoSWEET10R-2 under the stereomicroscope. Scale bar = 1 mm. (**B**) Longitudinal paraffin sections of mature *Arabidopsis* seeds of *atsweet10*, CoSWEET10R-1, and CoSWEET10R-2. Scale bar = 200 μm. (**C**) The 1000-grain weight of mature *Arabidopsis* seeds of WT, *atsweet10*, CoSWEET10R-1, and CoSWEET10R-2 (*n* = 3, three biological replicates). (**D**) The soluble sugar, sucrose, glucose, and fructose contents of WT, *atsweet10*, CoSWEET10R-1, and CoSWEET10R-2 in *Arabidopsis* siliques (*n* = 3, three biological replicates). (**E**) The TAG content of mature *Arabidopsis* seeds of WT, *atsweet10*, CoSWEET10R-1, and CoSWEET10R-2 (*n* = 3, three biological replicates). Each TAG content measurement used 200 seeds. The data represent the means ± SDs of three biological replicates. Significant differences from *atsweet10* of the corresponding group are indicated by asterisks (* *p* < 0.05) according to Student’s *t*-test.

Since CoSWEET10 was located on the plasma membrane and had high expression levels at late seed development, we speculated that CoSWEET10 could make a difference in seed development. The mature *Arabidopsis* seeds of *atsweet10*, R1, and R2 in the T_3_ generation were collected for further research. Compared to the mutant seeds, the seeds of R1 and R2 were more rounded and plumper, enriched with more inclusions (Figure 5A,B). The 1000-grain weights of both R1 and R2 were greater than that of *atsweet10*, where the R1 and R2 weights were 16% and 27% higher compared to *atsweet10*, respectively, and the *atsweet10* weight was 3.8% less compared with the WT (Figure 5C), suggesting CoSWEET10 can not only restore the phenotype of the *atsweet10* mutant but can also promote seed development. Then, we analyzed the total soluble sugar, sucrose, glucose, and fructose contents of the different lines. The content of total soluble sugar and sucrose of the CoSWEET10 transgenic restoration lines was greatly increased compared to *atsweet10* and WT, but not for glucose and fructose (Figure 5D). Triacylglycerol (TAG) is the major oil in *Arabidopsis* seeds, so we measured its content in different lines to see if the oil content changed. Compared to *atsweet10*, the TAG oil content of R1 and R2 increased by 16.7% and 22.9%, respectively (Figure 5E). These results indicate that CoSWEET10 contributes to the accumulation of soluble sugars and oil, thereby affecting seed development and yield formation.

### 2.7. CoSWEET10 Enhances Drought Tolerance in Transgenic Arabidopsis

**Figure 6:**
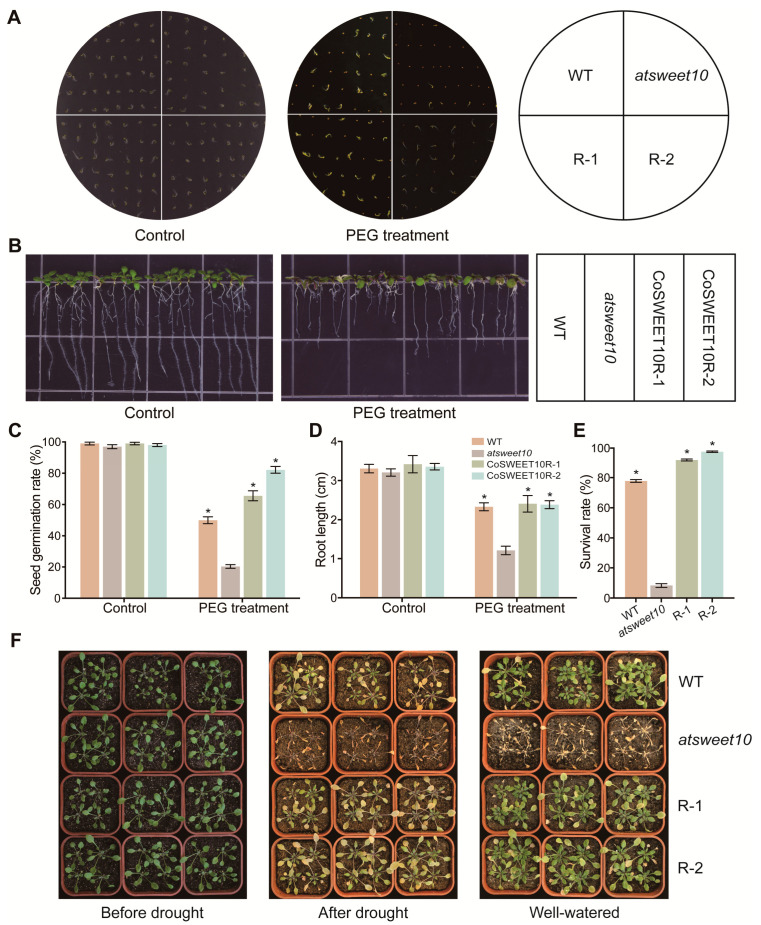
CoSWEET10 enhances drought tolerance in transgenic *Arabidopsis*. (**A**) The phenotypes of *Arabidopsis* seed germination of *atsweet10*, CoSWEET10R-1, and CoSWEET10R-2 on MS medium supplemented with PEG. *Arabidopsis* seeds grown on MS without PEG were used as a control. (**B**) The phenotypes of *Arabidopsis* seedlings of WT, *atsweet10*, CoSWEET10R-1, and CoSWEET10R-2 grown on 1/2 MS and 1/2 MS containing PEG. *Arabidopsis* seedlings grown on 1/2 MS without PEG were used as a control. (**C**) The *Arabidopsis* seed germination rates on MS (control) and MS containing PEG (*n* = 3, three biological replicates). (**D**) The root length of *Arabidopsis* seedlings grown on 1/2 MS (control) and 1/2 MS containing PEG (*n* = 4, four biological replicates). (**E**) The survival rate of *Arabidopsis* seedlings on the fifth day of re-watering (*n* = 4, four biological replicates). Plants were regarded as surviving if their leaves returned to green or new green leaves grew. The data represent the means ± SDs of at least three biological replicates. Significant differences from *atsweet10* under the same treatment are indicated by asterisks (* *p* < 0.05) according to Student’s *t*-test. (**F**) The phenotypes of *Arabidopsis* seedlings of WT, *atsweet10*, CoSWEET10R-1, and CoSWEET10R-2 cultured in the soil before drought stress, after drought stress, and after re-watering.

To reveal the potential function of CoSWEET10 in drought tolerance, seeds and seedlings of *Arabidopsis* were grown on a solid MS medium supplemented with 10% polyethylene glycol (PEG) to exert osmotic stress for plants. As a control, almost all of the seeds on the MS medium germinated, but on the PEG-MS, the seed germination rates of R1 and R2 were markedly higher than those of WT and *atsweet10*, where R2 was almost four-fold that of *atsweet10* (Figure 6A,C). Under normal treatment, the growth of all seedlings showed no obvious difference for all the lines. However, the growth of seedlings of all lines with PEG treatment was inhibited, especially for the mutant of *atsweet10*. The root length of R1 and R2 on the PEG-medium was significantly longer than that of *atsweet10*, in which R1 and R2 were both about two-fold that of *atsweet10*, though the length of *atsweet10* was about half that of WT (Figure 6B,D). We further subjected *Arabidopsis* seedlings under soil culture conditions to a 14 d-drought stress treatment. Leaves of *atsweet10* exhibited the most severe wilting and drooping, while R1 and R2 showed only slight phenotypic changes (Figure 6F). After 5 d of re-watering, almost all R1 and R2 lines and the majority of WT rejuvenated, but the *atsweet10* mutants did not (Figure 6E,F). These results indicate that CoSWEET10 can endow the seeds with greater vigor and enhance plant tolerance to adverse environments.

## 3. Discussion

SWEET transporters in plants are mostly involved in intercellular sugar transport, and contribute to the growth of sink organs by translocating carbohydrates from source leaves to sinks [37]. Phylogenetic analysis has shown that CoSWEET10 belongs to Clade III of the SWEET family and has the highest homology with *Arabidopsis AtSWEET10*, which also belongs to Clade III. It was reported that Clade III SWEETs preferred transport activity for sucrose over glucose and were unable to transport maltose [9]. In our present study, CoSWEET10, as a plasma membrane-localized protein, had a high expression level in tea seed oil and had the ability to transport sucrose, glucose, and fructose, which was demonstrated simultaneously in yeast cells and transgenic *Arabidopsis* (Figure 3 and Figure 4). AtSWEET10, the homolog of CoSWEET10 in *Arabidopsis*, is also located on the plasma membrane and functions as a sucrose transporter [9], suggesting that the substrate specificity of SWEETs is relatively conserved for the same clade among plant species. Of course, some functional variation also occurred with the SWEET members of plants since CoSWEET10 functioned as a specific transporter of sucrose, glucose, fructose, and galactose in this study. Similar phenomena have also been observed in other plants. The *GmSWEET15a* and *15b* in soybeans mediate the transport of sucrose from the endosperm to the embryo [38], while *GmSWEET10a* and *GmSWEET10b* influence the seed size and oil content by transporting both sucrose and glucose from the seed coat to the embryo [18]. SWEETs not only function as sugar transporters but also as sugar alcohol and hormone carriers [37]. For example, Clade III members AtSWEET13 and 14 are capable of transporting sucrose and are involved in gibberellic acid (GA) transport [39]. Here, we found that CoSWEET10 cannot transport mannose. It still needs to be explored whether CoSWEET10 functions as a hormone carrier.

Some SWEETs normally establish translocation pores by assembling into oligomers to transport sugars, especially large substrates such as sucrose [40]. In *Arabidopsis*, seventeen AtSWEET proteins can form 8 homo-oligomers and 47 hetero-oligomers [36]. In tomatoes, *SlSWEET14* belongs to Clade III and can form homo-oligomers and hetero-oligomers with *SlSWEET7a* in the split-GFP assay [41]. However, the homo-oligomers of CoSWEET10 were not observed in the split-GFP assay (Figure S2). It remains to verify in future research whether CoSWEET10 can form hetero-oligomers with other SWEETs. 

Previous studies have shown that sugars could be signal molecules to feedback regulate the expressions of SWEETs. In tomatoes, *SlSWEET14* expression could respond to glucose, and its promoter sequence was identified to include many sugar-responsive elements [41]. In maize, *ZmSWEET4c* expression could be induced by glucose and sucrose in the course of endosperm culture in vitro [15]. Our findings also demonstrate that the expression of CoSWEET10 could be induced by hexoses and sucrose in an in vitro *C. oleifera* seed culture, with glucose having the most apparent effects (Figure 3). This is further demonstrated in CoSWEET10 transgenic *Arabidopsis*. The *atsweet10* mutant showed an obvious root growth defect on 1.5% glucose-medium compared to the WT, R1, and R2 lines. However, on the medium with a high content of sucrose or glucose (4%), the seedling growth of the R1 and R2 lines was dramatically suppressed compared to *atsweet10*, which had the least suppression which was particularly obvious on the 4% glucose-medium (Figure 4). These results suggest that CoSWEET10 can restore the sugar transport in *atsweet10* and is more sensitive to glucose, which probably acts as a signal molecule to the feedback regulation of the expression of CoSWEET10.

SWEETs have been widely reported to be involved in the development of sink organs, especially fruits and seeds. In *Arabidopsis*, *AtSWEET11, 12*, and *15*, which are closely related to transporters of CoSWEET10, are located on the seed coat and work together in developing seeds [22]. The *sweet11;12;15* triple mutants exhibited severe seed defects, such as decreased seed weight, and a reduced starch and lipid content [22]. In maize, *ZmSWEET4c* plays a key role in enhancing sugar importation into the endosperm during the seed-filling period [15]. In soybeans, *GmSWEET10a* and *10b* are involved during seed development and have a significant effect on seed size and oil content [18]. Our results showed that CoSWEET10 had an influence on the seed development and accumulation of sugar and oil in plants. CoSWEET10 was able to restore the seed defect phenotype of *atsweet10*, producing more rounded and plumper seeds. Physiological detection showed that the content of total soluble sugar, sucrose, and oil was especially increased in CoSWEET10 transgenic lines (Figure 5). These results suggest that CoSWEET10 participates in seed development and yield formation mainly by affecting the accumulation of sugars and oil.

Recently, a growing body of evidence has shown that SWEETs are involved in adverse conditions such as biotic and abiotic stress [31,42,43,44]. In *Camellia sinensis*, *CsSWEET1a* and *CsSWEET17* are involved in improving the freezing tolerance of *Arabidopsis* [44]. *AtSWEET17* has a great abundance in the roots to transport fructose under cold and drought stress [31]. The transcript level of *AtSWEET10* significantly increased in the waterlogging condition, which led to ethylene formation [45]. In this study, we also found that CoSWEET10 enhanced the drought tolerance of *Arabidopsis* in transgenic lines. On one hand, the CoSWEET10 transgenic lines had higher seed germination and longer roots under PEG treatment, suggesting CoSWEET10 makes seeds more vigorous and strong in response to drought stress. On the other hand, mature CoSWEET10 transgenic seedlings in the soil are more tolerant under drought treatment. Physiological detection showed that the transgenic *Arabidopsis* lines of CoSWEET10 accumulated higher concentrations of soluble sugars, especially more sucrose compared to the WT and *atsweet10* mutant, which probably served as osmoprotectants to improve plants’ drought tolerance. In *Arabidopsis*, *AtSWEET11* and *AtSWEET12* enhanced transcript abundance under drought conditions and promoted carbohydrate import from source leaves into roots in response to drought stress [33]. High levels of soluble sugars not only influence the development and yield of plants but also improve plant abiotic tolerance by functioning as osmoprotectants to maintain the architecture of the cell membrane [28,29]. In addition, abscisic acid (ABA) serves as a signaling molecule involved in multiple stress responses in plants. ABA was also reported to take part in SWEET-mediated drought resistance. For instance, in rice, *OsSWEET13* and *15* strongly express under both drought and ABA treatment [32]. The mechanism of increased activities of *AtSWEET11* and *12* under drought conditions is also correlated with ABA [46]. ABA signaling induced by the drought treatment leads to the activation of SnRK2s, which enhances the oligomerization of *AtSWEET11* and *12*, resulting in a sharp increase in sucrose transport activity and root biomass, ultimately improving plant drought tolerance [46]. However, CoSWEET10 might not improve plant drought tolerance through the ABA signaling pathway because there are no ABA response elements in the promoter region of CoSWEET10, but an MYB-binding site involved in drought-inducibility is contained (Table S4). Therefore, we speculate that CoSWEET10 may be regulated by MYB to influence plant drought tolerance, however, its specific drought tolerance mechanism needs to be verified in further research.

## 4. Materials and Methods

### 4.1. Plant Materials and Growth Conditions

All samples were picked from *C. oleifera* ‘Huashuo’, which grew in Changsha, Hunan province, China. The pollination period began around November, samples were collected from 170 DAP (days after pollination) to 320 DAP and immediately frozen in liquid nitrogen and stored at −80 °C for further research. 

An *Arabidopsis atsweet10* (homolog of CoSWEET10) mutant was ordered from ABRC (*Arabidopsis* Biological Resource Center). The *Arabidopsis* restoration lines were generated by genetically transforming the 35S::CoSWEET10 resulting constructs into the mutant plants. Seeds from T_3_ homozygous generations were utilized for further research. *Arabidopsis* WT (wild-type) plants were used as a control. For the functional assays, *Arabidopsis* seeds were first sown on the MS medium in the dark condition at 4 °C for 3 d, then moved to the greenhouse, and lastly, the seedlings were transplanted on the 1/2 MS supplemented with different sugars. For the drought treatment, *Arabidopsis* seeds were sowed on the MS medium containing 10% PEG6000 (polyethylene glycol) [47,48] and treated as described above until the seeds germinated, or seeds were sown on the MS medium as described above, then the seedlings were transplanted on the 1/2 MS containing 10% PEG or to the soil mixture consisting of 2:1 nutrient soil:vermiculite. Seedlings on the MS (or 1/2 MS) medium without PEG were used as a control. Seedlings on the soil mixture experienced two steps, including a 14 d drought treatment and 5 d re-watering. The greenhouse had a 16 h/8 h (light/dark) photoperiod at 25 °C and a relative humidity of 50–70%.

### 4.2. Protein Sequence and Phylogenetic Analysis

The SWEET protein sequences of different plant species were obtained via BLAST against the CoSWEET10 gene sequence on NCBI (https://www.ncbi.nlm.nih.gov, accessed on 7 June 2022). The CoSWEET10 gene sequence is listed in Supplementary Table S1. The conserved MtN3/saliva domain position was obtained on the NCBI conserved domain database (CDD) (https://www.ncbi.nlm.nih.gov/Structure/cdd/wrpsb.cgi, accessed on 17 June 2022). The alignment of protein sequences was accomplished on ESPript 3.0 (http://espript.ibcp.fr/ESPript/cgi-bin/ESPript.cgi, accessed on 28 June 2022). The phylogenetic tree of CoSWEET10 and AtSWEETs was completed with MEGA software and the neighbor-joining method. The amino acid sequences used for performing this phylogenetic analysis are listed in Supplementary Table S2.

### 4.3. RNA Extraction and qRT-PCR (Quantitative Real-Time PCR) Experiments

Total RNA was extracted from different tissues, including the flowers, stems, young leaves, mature leaves, and fruits of *C. oleifera* ‘Huashuo’ using the OminiPlant RNA Kit (DNase I) (CWBIO, Beijing, China). First-strand cDNA was synthesized using the AccuRT Genomic DNA Removal Kit (Applied Biological Materials Inc., Vancouver, BC, Canada). qRT-PCR was performed with SYBR Green Master Mix enzymes (ABI, Vernon, CA, USA), and the relative quantification of transcript levels was analyzed by the cycle threshold 2^−∆∆CT^ method [49]. The *C. oleifera GAPDH* gene was as an internal control. The primer sequences are listed in Supplementary Table S3.

### 4.4. Subcellular Localization of CoSWEET10

The coding sequence of the CoSWEET10 protein was C-terminally synthesized in the pSuper1300-GFP vector, which was driven by the CaMV35S promotor. The resulting constructs were transformed into the *A. tumefaciens* strain GV3101 and then used to infect the epidermal cells of *N. benthamiana* leaves. pBI121-mCherry-fABD2 (Red Fluorescent Protein, RFP) acted as a plasma membrane marker [50]. Two days after infection, the fluorescence signals were observed using a confocal laser scanning microscope (Carl Zeiss LSM780) with acquisition at 440–500 nm (GFP) and 540–600 nm (RFP). Experiments were performed three times independently.

### 4.5. Bimolecular Fluorescence Complementation Assays

The coding sequence for the CoSWEET10 protein was synthesized in the P2YC (cYFP) and P2YN (nYFP), respectively. The whole vectors were then transformed into the *A. tumefaciens* strain GV3101. Subsequently, the fusion proteins were separately transformed or co-transformed into epidermal cells of tobacco leaves. Two days after infection, the fluorescence signals were observed using a confocal laser scanning microscope (Carl Zeiss LSM780) with acquisition at 510 nm and 550 nm. 

### 4.6. Complementation Assays for CoSWEET10 in Yeast Cells

For the complementation assay in yeast (*Saccharomyces cerevisiae*) cells, the CDS of CoSWEET10 and AtSUC2 were cloned into the yeast expression vector pDR196. The empty pDR196 vector was used as a negative control and the AtSUC2-pDR196 construct was used as positive sucrose transporter control; the resulting constructs were transformed into the yeast mutant strain EBY.VW4000 (hexose transport-deficient strain) and SUSY7/ura3 (sucrose uptake-deficient strain), respectively. Transformants of the EBY.VW4000 were grown in liquid SD/−Ura media containing 2% maltose as the sole carbon source, while 2% glucose was used for the SUSY7/ura3. Serial dilutions of yeast cell suspensions (10, 100, and 1000-fold values) of EBY.VW4000 were dropped on solid SD/−Ura media with 2% maltose or 2% glucose/fructose/galactose/mannose as the sole carbon source, while 2% glucose or 2% sucrose was used for the SUSY7/ura3. Yeast cells were grown at 30 °C for 3–4 days.

### 4.7. C. oleifera Seed In Vitro Culture on Medium Containing Different Sugars

*C. oliefera* seeds at 230 DAP were disinfected with 75% alcohol for 45 s followed by immersion in 1% corrosive sublimate for 10 min. They were then placed on a solid medium with 3% sucrose, 3% glucose, or 3% fructose. The solid media with no sugar was used as a control. There were around 10 seeds in each plastic petri dish. After 4 d or 8 d in the dark at 24 °C, seeds with the same treatment were collected and immediately frozen in liquid nitrogen and stored at −80 °C. RNA was extracted from these samples as described above. Each treatment was performed in three biological replicates. The total experiment process is referred to Sosso et al [15].

### 4.8. Paraffin Embedding and Sectioning

Freshly collected *Arabidopsis* seeds were placed into a fixative solution of Carnoy (formaldehyde:ethanol = 3:1) for 24 h, then dehydrated with an increasing ethanol gradient (70%, 80%, 90%, 95%, and 100%), infiltrated with a xylene/ethanol series (ethanol/xylene 1:1, and 100% xylene), and next permeated with a xylene/paraffin series (xylene/paraffin 3:1, and 100% wax solution) for 12 h; the seeds were finally embedded with a wax solution in cartons. Semithin longitudinal sections were cut by rotary microtome (Leica RM2265, Berlin, Germany). After deparaffinizing and hydrating with a xylene/ethanol series, sections were observed using a biological microscope (Leica DM 2500, Berlin, Germany).

### 4.9. Sugar Quantification of Arabidopsis Siliques

Mature *Arabidopsis* siliques were used for sugar concentration measurements. Sucrose, glucose, fructose, and whole soluble sugar were extracted and then analyzed via high-performance liquid chromatography (HPLC) as described previously [51,52,53]. Each assay was performed in three biological replicates.

### 4.10. Oil Content Measurement of Arabidopsis Seeds

Triacylglycerol (TAG) is the major oil in *Arabidopsis* seeds, so we used its content to represent oil content. A total of 200 dry and mature *Arabidopsis* seeds were used for one TAG content measurement. The extraction and measurement of TAG were performed as described previously [54,55]. Each assay was performed in three biological replicates.

### 4.11. Statistical Analysis

All data were analyzed by SPSS software 16.0 and graphed with GraphPad Prism 9.0. The data are presented as the means ± SDs of at least three replicates. Significance tests were determined at *p* < 0.05 based on Student’s *t*-tests.

## 5. Conclusions

CoSWEET10 is a plasma membrane-localized protein and has great abundance in the seeds of *Camellia oleifera*. CoSWEET10 belongs to Clade III and can transport sucrose, glucose, and fructose. As a sucrose and hexose transporter, the expression of CoSWEET10 is also regulated by its transport substrate, especially glucose. The transgenic *Arabidopsis* lines of CoSWEET10 accumulated more sucrose and soluble sugars and restored the seed defect phenotype of *atsweet10*. In addition, CoSWEET10 transgenic plants showed more tolerance under drought treatment during the seed and seedling stages. Our study provides evidence that CoSWEET10 plays a dual role in promoting seed development and yield formation and enhancing the drought tolerance of plants. 

## Data Availability

All relevant data can be found within the manuscript and its supporting material.

## Supplementary Materials

The following supporting information can be downloaded at: https://www.mdpi.com/article/10.3390/plants12152818/s1, Table S1: Coding sequence (CDS) of *CoSWEET10*, Table S2: The amino acid sequences used for constructing the phylogenetic tree, Table S3: The primers used in this study, Table S4: Analysis of the cis-acting elements of the CoSWEET10 promoter; Figure S1: Protein sequence alignment of AtSWEETs and CoSWEET10, Figure S2: Bimolecular fluorescence complementation assay of self-interaction of CoSWEET10, Figure S3: Identification of mutant and restored lines in *Arabidopsis.*
