# Supplementary material for: Camellia oleifera CoSWEET10 Is Crucial for Seed Development and Drought Resistance by Mediating Sugar Transport in Transgenic Arabidopsis
Source: Plants (Basel). 2023 Jul 29;12(15):2818. doi: 10.3390/plants12152818 (PMC10420866; doi:10.3390/plants12152818)
Supplement: Supplementary file 1 [file plants-12-02818-s001.zip › plants-2468592-supplementary.pdf]

Table S1. Coding sequence (CDS) of *CoSWEET10*

Gene name	CDS
CoSWEET10	ATGGGTGGGTTCTCTAGTCACAATTTGGCTTTCGCATTTGGCCTTTTAGGCAACATCATCTCATTCTTTGTCTTCCTTTCACCCCTGCCAACATTTTACCAA<br>ATATTCAAGAAAAAATCAACTGAAGGGTTCCAATCGGTTCCTTACATAGTTGCCTTGTTTAGTGCTATGTTGTTGATGTATTACGCATTTCTCAAGAAGAT<br>TGACTCTACACTTATCATCACTATAAACTCATTTGGATGCTTGGTCGAAACTATTTATATTTGTGTTTATCTCTTTTACGCTCCCAAGAAGATTAAGTTCAAA<br>ACCATGAAACTACTTGGATTCATGCTTGGCGGGTTTGGCGCGATCCTTATCTTAACTCAATTTCTTATCAAAAAAAGCTCAACCCGCTTTCACATTGTTGG<br>TTGGATTTGCCTATTGTTCTCTGTGAGCGTTTTCGCCGCTCCTCTCTGCATATTGAAACAAGTGATACGAACAAAGAGCGTAGAGTTCATGCCATTTTCCT<br>TATCACTTTCGCTCACGCTTAACGCGGTCATGTGGTTCTTCTATGGCCTTCTAATAAAAGACTTCAACATTGCTATTCCGAATGTCTTGGGGTTTATTTTTG<br>GAATTATTCAAATGGTGCTTTACGCGATATACAAGAACCCAAAGAAAGTTGTTGCGAAAGATCAAAAGTTTTCTTCTGATCAAATACCAAACAAAGTGA<br>TAGCCTTGGAGGAAGAGAAGCTATCTGAATTAGTTGAACAAGTGATCGATGTTGTGAAAGATCAAAAGTTTTCTTCTGATCAAATACCAAACAAAGTCA<br>TAGCCTTGGAGGAAGAGAAGCTATCTGAATTAGTCGAACAAGTGATTGATGTTGTGAAGCTTAGCTCAATCGCATGTCAAGAAATCGTTCCAGTCATGC<br>CTCATCTGAATGGACACAACATAGCTAGAGAGCTCAATACAATTAAACCAAACCTTGAAGTCACCGTGCCGGTCTAA

Table S2. The amino acid sequences used for constructing phylogenetic tree.

Protein name	Amino acid sequence
CoSWEET10	MGGFSSHNLAFAFGLLGNIISFFVFLSPLPTFYQIFKKKSTEGFQSVPYIVALFSAMLLMYYAFLKKIDSTLIITINSFGCLVETIYICVYLFYAPKKIKFKTMKLLG<br>FMLGGFGAILILTQFLIKKSSTRFHIVGWICLLFSVSVFAAPLCILKQVIRTKSVEFMPFSLSLSLTLNAVMWFFYGLLIKDFNIAIPNVLGFIFGIIQMVLYAIYKN<br>PKKVVAKDQKFSSDQIPNKVIALEEEKLSELVEQVIDVVKDQKFSSDQIPNKVIALEEEKLSELVEQVIDVVKLSSIACQEIVPVMPHLNGHNIARELNTIKPNL<br>EVTVPV
AtSWEET1	MNIAHTIFGVFGNATALFLFLAPSITFKRIIKNKSTEQFSGIPYPMTLLNCLLSAWYGLPFVSKDNTLVSTINGTGAVIETVYVLIFLFYAPKKEKIKIFGIFSCVLA<br>VFATVALVSLFALQGNGRKLFCGLAATVFSIIMYASPLSIMRLVVKTKSVEFMPFFLSLFVFLCGTSWFVYGLIGRDPFVAIPNGFGCALGTLQLILYFIYCGNKG<br>EKSADAQKDEKSVEMKDDEKKQNVVNGKQDLQV
AtSWEET2	MDVFAFNASLSMCKDVAGIAGNIFAFGLFVSPMPTFRRIMRNKSTEQFSGLPYIYALLNCLICLWYGTPFISHSNAMLMTVNSVGATFQLCYIILFIMHTDKKN<br>KMKMLGLLFVVFAVVGVIVAGSLQIPDQLTRWYFVGFLSCGSLVSMFASPLFVINLVIRTKSVEFMPFYLSLSTFLMSASFLLYGLFNSDAFVYTPNGIGTILGIV<br>QLALYCYYHRNSIEEETKEPLIVSYV
AtSWEET3	MGDKLRLSIGILGNGASLLLYTAPIVTFSRVFKKKSTEEFSCFPYVMTLFNCLIYTWYGLPIVSHLWENLPLVTINGVGILLESIFIFIYFYYASPKEKIKVGVTFV<br>PVIVGFGLTTAISALVFDDHRHRKSFVGSVGLVASISMYGSPLVVMKKVIETRSVEYMPFYLSFFSFLASSLWLAYGLLSHDLFLASPNMVATPLGILQLILYFKY<br>KNKKDLAPTTMVITKRNDHDDKNKATLEFVVDVDRNSDTNEKNSNNASSI
AtSWEET4	MVNATVARNIAGICGNVISLFLFLSPIPTFITIYKKKKVEEYKADPYLATVLNCALWVFYGLPMVQPDSLLVITINGTGLAIELVYLAIFFFFSPTSRKVKVGLWL<br>IGEMVFVGIVATCTLLLFHTHNQRSSFVGIFCVIFVSLMYIAPLTIMSKVIKTKSVKYMPFSLSLANFLNGVVWVIYALIKFDLFILIGNGLGTVSGAVQLILYAC<br>YYKTTPKDDEDEEDEENLSKVNSQLQLSGNSGQAKRVSA
AtSWEET5	MTDPHTARTIVGIVGNVISFGLFCAPIPTMVKIWKMKSVSEFKPDPYVATVLNCMMWTFYGLPFVQPDSLLVITINGTGLFMELVYVTIFFVFATSPVRRKITIA<br>MVIEVIFMAVVIFCTMYFLHTTKQRSMLIGILCIVFNVIMYAAPLTVMKLVIKTKSVKYMPFFLSLANFMNGVVWVIYACLKFDPYILIPNGLGSLSGIIQLIIYIT<br>YYKTTNWNDDDEDKEKRYSNAGIELGQA
AtSWEET6	MVHEQLNLIRKIVGILGNFISLCLFLSPTPTFIHIVKKKSVEKYSPLPYLATLLNCLVRALYGLPMVHPDSTLLVTISGIGITIEIVFLTIFFVFCGRQQHRLVISAVL<br>TVQVVFVATLAVLVLTLEHTTDQRTISVGIVSCVFNAMMYASPLSVMKMVIKTKSLEFMPFLLSVVGFLNAGVWTIYGFVPFDPFLAIPNGIGCVFGLVQLILY<br>GTYYKSTKGIMEERKNRLGYVGEVGLSNAIAQTEPENIPYLNKRVSGV
AtSWEET7	MVFAHLNLLRKIVGIIGNFIALCLFLSPTPTFVRIVKKKSVEEYSPIPYLATLINCLVWVLYGLPTVHPDSTLVITINGTGILIEIVFLTIFFVYCGRQKQRLIISAVIA<br>AETAFIAILAVLVLTLQHTTEKRTMSVGIVCCVFNVMMYASPLSVMKMVIKTKSVEFMPFWLSVAGFLNAGVWTIYALMPFDPFMAIPNGIGCLFGLAQLILY<br>GAYYKSTKRIMAERENQPGYVGLSSAIARTGSEKTANTNQEPNNV
AtSWEET8	MVDAKQVRFIIGVIGNVISFGLFAAPAKTFWRIFKKKSVEEFSYVPYVATVMNCMLWVFYGLPVVHKDSILVSTINGVGLVIELFYVGVYLMYCGHKKNHRR<br>NILGFLALEVILVVAIILITLFALKGDFVKQTFVGVICDVFNIAMYGAPSLAIIKVVKTKSVEYMPFLLSLVCFVNAGIWTTYSLIFKIDYYVLASNGIGTFLALSQ<br>LIVYFMYYKSTPKEKTVKPSEVEISATERV
AtSWEET9	MFLKVHEIAFLFGLLGNIVSFGVFLSPVPTFYGIYKKKSSKGFQSIPYICALASATLLLYYGIMKTHAYLIISINTFGCFIEISYLFLYILYAPREAKISTLKLIVICNI<br>GGLGLLILLVNLLVPKQHRVSTVGWVCAAYSLAVFASPLSVMRKVIKTKSVEYMPFLLSLSLTLNAVMWFFYGLLIKDKFIAMPNILGFLFGVAQMILYMMY

	QGSTKTDLPTENQLANKTDVNEVPIVAVELPDVGSDNVEGSVRPMK
AtSWEET10	MAISQAVLATVFGILGNIISFFVCLAPIPTFVRIYKRKSSEGYQSIPYVISLFSAMLWMYYAMIKKDAMMLITINSFAFVVQIVYISLFFFYAPKKEKTLTVKFVLF<br>VDVLGFGAIFVLTYFIIHANKRVQVLGYICMVFALSVFVAPLGIIRKVIKTKSAEFMPFGLSFFLTLSAVMWFFYGLLLKDMNIALPNVLGFIFGVLQMILFLIYK<br>KPGTKVLEPPGIKLQDISEHVVDVVRLSTMVCNSQMRTLVPQDSADMEATIDIDEKIKGDIEKNKDEKEVFLISKN
AtSWEET11	MSLFNTENTWAFVFGLLGNLISFAVFLSPVPTFYRIWKKKTTEGFQSIPYVVALFSATLWLYYATQKKDVFLLVTINAFGCFIETIYISMFLAYAPKPARMLTVK<br>MLLLMNFGGFCAILLLCQFLVKGATRAKIIGGICVGFSVCVFAAPLSIIRTVIKTRSVEYMPFSLSLTLTISAVIWLLYGLALKDIYVAFPNVLGFALGALQMILYV<br>VYKYCKTSPHLGEKEVEAAKLPEVSLDMLKLGTVSSPEPISVVRQANKCTCGNDRRAEIEDGQTPKHGKQSSSAAAT
AtSWEET12	MALFDTHNTWAFVFGLLGNLISFAVFLSPVPTFYRICKKKTTEGFQSIPYVVALFSAMLWLYYATQKKDVFLLVTINSFGCFIETIYISIFVAFASKKARMLTVKL<br>LLLMNFGGFCLILLLCQFLAKGTTRAKIIGGICVGFSVCVFAAPLSIIRTVIKTKSVEYMPFSLSLTLTISAVIWLLYGLALKDIYVAFPNVIGFVLGALQMILYVV<br>YKYCKTPSDLVEKELEAAKLPEVSIDMVKLGTLTSPEPVAITVVRSVNTCNCNDRNAEIENGQGVRNSAATT
AtSWEET13	MALTNNLWAFVFGILGNIISFVVFLAPVPTFVRICKKKSTEGFQSLPYVSALFSAMLWIYYAMQKDGTAFLLITINAFGCVIETIYIVLFVSYANKKTRISTLKVL<br>GLLNFLGFAAIVLVCELLTKGSTREKVLGGICVGFSVSVFAAPLSIMRVVVRTRSVEFMPFSLSLFLTISAVTWLFYGLAIKDFYVALPNVLGAFLGAVQMILYII<br>FKYYKTPVAQKTDKSKDVSDHSIDIAKLTTVIPGAVLDSAVHQPPALHNVPETKIQLTEVKSQNMTDPKDQINKDVQKQSQV
AtSWEET14	MVLTHNVLAVTFGVLGNIISFIVFLAPVPTFVRICKKKSIEGFESLPYVSALFSAMLWIYYALQKDGAGFLLITINAVGCFIETIYIILFITYANKKARISTLKVLGL<br>LNFLGFAAIILVCELLTKGSNREKVLGGICVGFSVCVFAAPLSIMRVVIRTKSVEFMPFSLSLFLTISAITWLFYGLAIKDFYVALPNILGAFLGAVQMILYVIFKY<br>YKTPLVVDETEKPKTVSDHSINMVKLSSTPASGDLTVQPQTNPDVSHPIKTHGGDLEDQMDKKMPN
AtSWEET15	MGVMINHHFLAFIFGILGNVISFLVFLAPVPTFYRIYKRKSTESFQSLPYQVSLFSCMLWLYYALIKKDAFLLITINSFGCVVETLYIAMFFAYATREKRISAMKLF<br>IAMNVAFFSLILMVTHFVVKTPPLQVSVLGWICVAISVSVFAAPLMIVARVIKTKSVEYMPFTLSFFLTISAVMWFAYGLFLNDICIAIPNVVGFVLGLLQMVLY<br>LVYRNSNEKPEKINSSEQQLKSIVVMSPLGVSEVHPVVTESVDPLSEAVHHEDLSKVTKVEEPSIENGKCYVEATRPETV
AtSWEET16	MADLSFYVGVIGNVISVLVFLSPVETFWRIVQRRSTEEYECFPYICTLMSSSLWTYYGIVTPGEYLVSTVNGFGALAESIYVLIFLFFVPKSRFLKTVVVVLALN<br>VCFPVIAIAGTRTLFGDANSRSSSMGFICATLNIIMYGSPLSAIKTVVTTRSVQFMPFWLSFFLFLNGAIWGVYALLLHDMFLLVPNGMGFFLGIMQLLIYAYYR<br>NAEPIVEDEEGLIPNQPLLA
AtSWEET17	MAEASFYIGVIGNVISVLVFLSPVETFWKIVKRRSTEEYKSLPYICTLLGSSLWTYYGIVTPGEYLVSTVNGFGALVETIYVSLFLFYAPRHLKLKTVDVEAML<br>NVFFPIAAIVATRSAFEDEKMRSQSIGFISAGLNIIMYGSPLSAMKTVVTTKSVKYMPFWLSFFLFLNGAIWAVYALLQHDVFLLVPNGVGFVFGTMQLILYGIY<br>RNAKPVGLSNGLSEIAQDEEEGLTSRVEPLLS

Table S3. The primers used in this study.

Gene name	Forward primer (5'-3')	Reverse primer (5'-3')	Purpose
CoSWEET10	ATGGGTGGGTTCTCTAGTCACAAT	TTAGACCGGCACGGTGACT	Gene clone
	GTTGTTGCGAAAGATCAAAAGTT	TTCTTGACATGCGATTGAGCTAA	qRT-PCR
GADPH	GGTGCCAAGAAGGTGGTAATA	GTTGTGCAGCTTGCATTAGAG	qRT-PCR
CoSWEET10	GGGGCCCGGGGTCGACATGGGTG GGTTCTCTAG	CCATGGTACCGGATCCGACCGGC ACGGTGAC	Subcellular localization assays
	CGAGCTCAACTTCGAAATGGGTG GGTTCTCTAG	TTCCCTCGAGGTCGACTTAGACC GGCACGGTG	Yeast complementation assays
	CGCCACTAGTGGATCCATGGGTGG GTTCTCTAG	TCCCGGGAGCGGTACCGACCGG CACGGTGAC	BiFC assays (cYFP)
	CGCCACTAGTGGATCCATGGGTGG GTTCTCTAG	TCCCGGGAGCGGTACCGACCGG CACGGTGAC	BiFC assays (nYFP)
	AATACTAGTGGATCCGGTAC	GATGAACTTCAGGGTCAGCT	*Arabidopsis* transgenic restoration lines generation

Table S4. Analysis of cis-acting elements of *CoSWEET10* promoter

Cis-acting element	Sequence	function	Number of copies
ARE	AAACCA	cis-acting regulatory element essential for the anaerobic induction	3
AE-box	AGAAACTT	part of a module for light response	2
TGA-element	AACGAC	auxin-responsive element	1
CAAT-box	CAAT/CAAAT	common cis-acting element in promoter and enhancer regions	25
TATA-box	TATA	core promoter element around -30 of transcription start	142
GC-motif	CCCCCG	enhancer-like element involved in anoxic specific inducibility	1
G-box	CACGAC	cis-acting regulatory element involved in light responsiveness	1
Box 4	ATTAAT	part of a conserved DNA module involved in light responsiveness	1
CGTCA-motif	CGTCA	cis-acting regulatory element involved in the MeJA-responsiveness	2
TGACG-motif	TGACG	cis-acting regulatory element involved in the MeJA-responsiveness	2
MBS	CAACTG	MYB binding site involved in drought-inducibility	1
MYC	CATGTG/CATTTG	MYC binding site	4
I-box	GATAA	part of a light responsive element	1

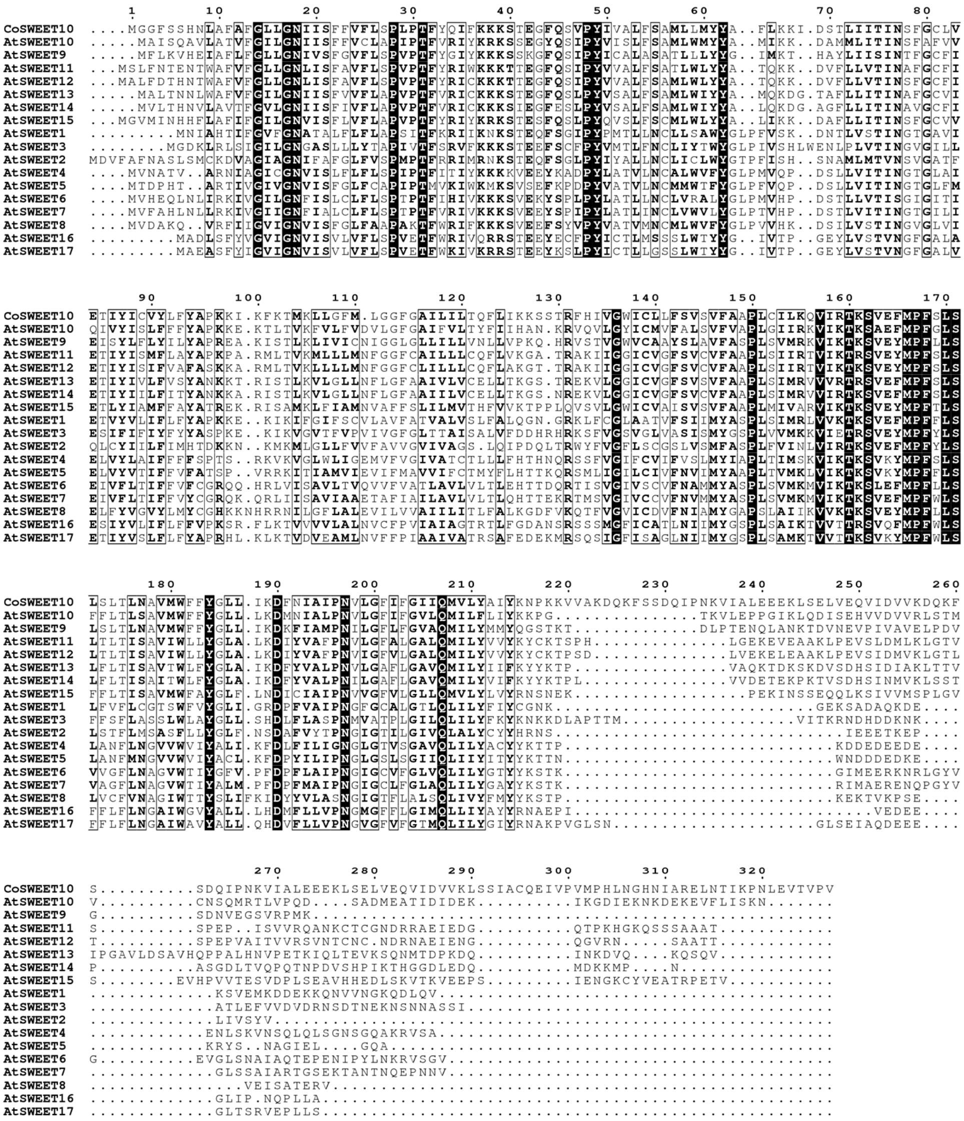

Figure S1. Protein sequence alignment of AtSWEETs and CoSWEET10.

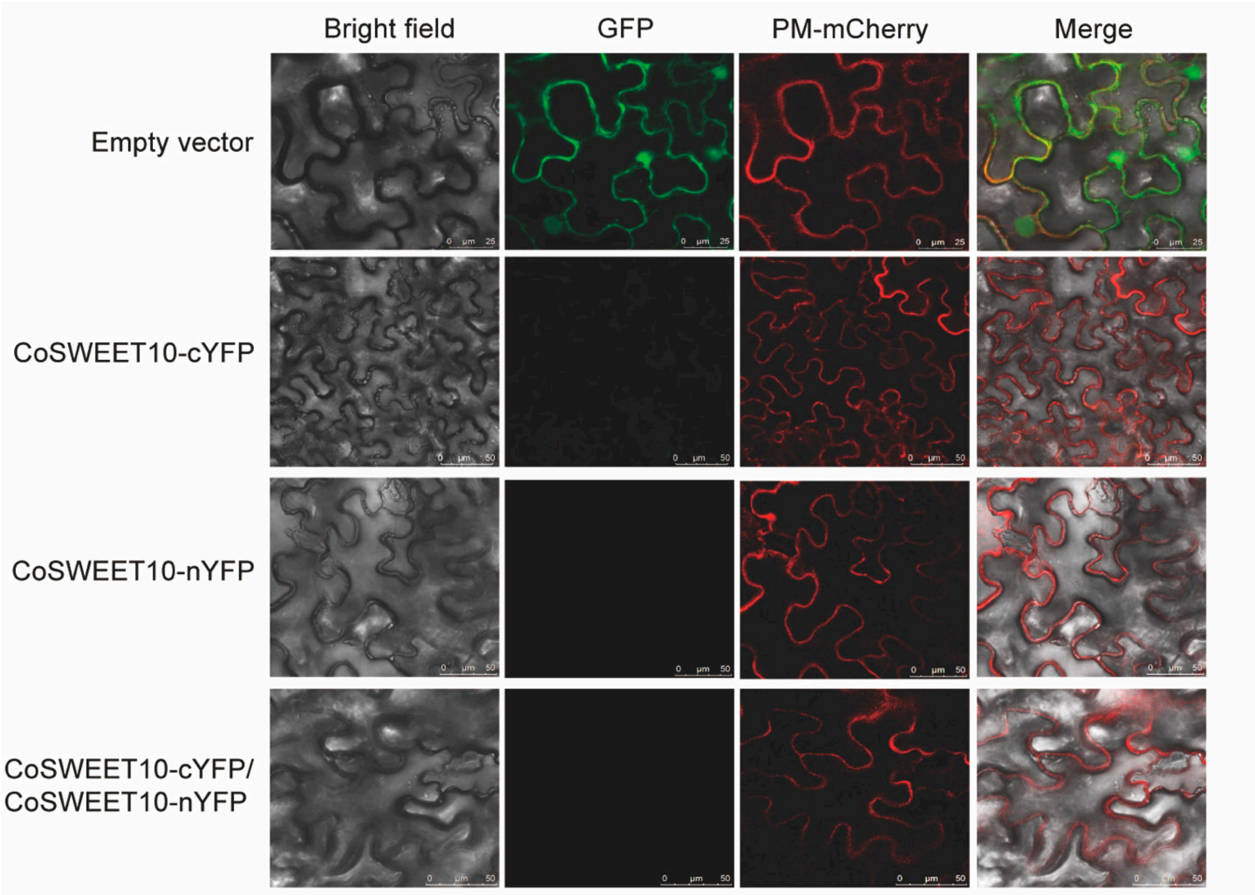

Figure S2. Bimolecular fluorescence complementation assay of self-interaction of CoSWEET10. Scale bars = 25 μm.

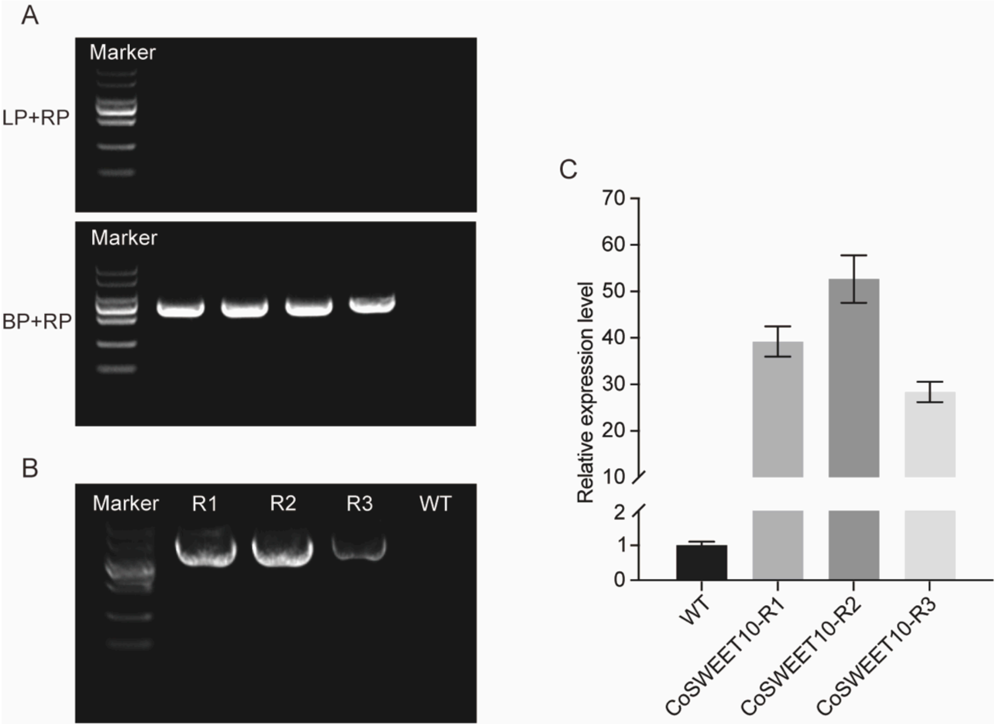

Figure S3. Identification of mutant and restored lines of *Arabidopsis.* (A) Electrophoretic image of PCR products of atsweet10 mutant. (B) Electrophoretic image of PCR products of WT and restoration lines. (C) Relative expression levels of CoSWEET10 in WT and restoration lines of *Arabidopsis.* Transcript levels were determined by qRT-PCR and relative to GAPDH. The expression data of WT was normalized to 1. The data are shown as the means ± SDs of three replicates.
